# ASO Author Reflections: Serum IgA as a Predictive Biomarker for Immunotherapy in Esophageal Squamous Cell Carcinoma

**DOI:** 10.1245/s10434-026-20003-w

**Published:** 2026-06-17

**Authors:** Kosaku Torii, Akimitsu Iizuka, Mitsuro Kanda

**Affiliations:** Department of Gastroenterological Surgery, Nagoya University Graduate School of Medicine, Nagoya, Japan

## Past

Esophageal squamous cell carcinoma (ESCC) is frequently diagnosed at advanced stages and demonstrates a high rate of recurrence even after curative resection.^1^ In Japan, current clinical practice guidelines recommend the use of immune checkpoint inhibitors (ICIs) as a first-line therapy for unresectable ESCC. However, early disease progression after the initiation of ICI therapy has emerged as a major clinical concern, underscoring the urgent need for reliable predictive biomarkers.

While Immunoglobulin A (IgA) is well recognized as the principal antibody mediating mucosal immunity in the gastrointestinal and respiratory tracts, about 10 % of IgA circulates in the bloodstream and contributes to systemic immune responses.^2^ However, few studies have examined the relationship between serum IgA levels and systemic therapy for solid tumors, and reports linking serum IgA to the efficacy of ICIs are particularly scarce. To date, no study has investigated the association between serum IgA levels and ICI treatment outcomes in ESCC.

## Present

In this study, we evaluated the association between pretreatment serum IgA levels and survival outcomes in patients with unresectable ESCC receiving first-line ICI therapy. The overall response rate was 10% in the high IgA group, compared with 70% in the low IgA group. Furthermore, progression-free survival (PFS) was significantly shorter in the high IgA group than in the low IgA group.^3^

Serum IgA offers several distinct advantages as a clinical tool: it is minimally invasive, low-cost, easily accessible using routine blood samples, and feasible for repeated assessment throughout the treatment course. Our findings demonstrate that pretreatment serum IgA levels can serve as a novel and practical predictive biomarker for unresectable ESCC, potentially optimizing patient selection for immunotherapy.

## Future

Our study is limited by a small sample size of 20 patients and a relatively short observation period. Therefore, future large-scale studies with extended follow-up are warranted to validate our findings. On the basis of previous literature,^4^ we speculate that high serum IgA levels may indirectly reflect an immunosuppressive tumor microenvironment (TME) which contributes to poor ICI efficacy; however, this remains a hypothesis. Further histopathological and immunological investigations are required to elucidate the underlying mechanisms within the TME, ultimately paving the way for personalized immunotherapy strategies in advanced ESCC.
